# Thanatophoric dysplasia: a case report

**DOI:** 10.11604/pamj.2020.37.220.21211

**Published:** 2020-11-05

**Authors:** Olusoji Edward Jagun, Mojisola Adejoke Olusola-Bello, Abiodun Folashade Adekanmbi, Omodele Oluyemisi Jagun, Tolani Oduwole

**Affiliations:** 1 Department of Obstetrics and Gynaecology, Olabisi Onabanjo University Teaching Hospital, Olabisi Onabanjo University Sagamu, Ogun state, Nigeria,; 2 Department of Radiology Olabisi Onabanjo University Sagamu, Sagamu, Nigeria,; 3 Department of Paediatrics, Olabisi Onabanjo University Teaching Hospital Sagamu, Sagamu, Nigeria,; 4 Department of Ophthamology, Babcock University Teaching Hospital, Ilishan, Nigeria,; 5 Alpha Clinic and Ultrasound Unit, Sagamu, Ogun state, Nigeria

**Keywords:** Fetal death, thanatophorism, skeletal dysplasia, ultrasound diagnosis

## Abstract

A case of thanatophoric dysplasia with sudden death at term is hereby presented. Thanatophoric dysplasia is an uncommon, lethal skeletal dysplasia which is associated with mutation in the extracellular region of fibroblast growth factor receptor 3 (FGFR3). It is an autosommal dominant condition that has sporadic occurrence and early ultrasound scan has not been of great benefit in its detection. Diagnosis is mostly made in the third trimester. The fetal death is usually due to severe respiratory insufficiency from a reduced thoracic capacity and hypoplastic lungs and/or respiratory failure due to brainstem compression. In view of the autosomal dominance of TD, it will be advisable for a woman with previous history to have prenatal screening to relieve parental anxiety and prevent late detection.

## Introduction

The detection of congenital malformations in the fetus whether in utero or at birth continue to be a sad moment for the physician and the parents. The increasing proficiency in ultrasound scanning and the accessibility has made detection rate to be high and offer of early termination of pregnancy is given in some cases. However, the detection of some structural abnormalities will remain elusive until much later when they can be diagnosed. Some are compatible with life and the others are not, generally mortality is very high among major congenital anomalies with the low- and middle-income countries having between 20-85% as against less than 10% in developed countries [1], only accurate diagnosis can assist in determining how best to manage the patient. Thanatophoric dysplasia (TD) is a rare congenital anomaly whose incidence is said to be about 1 in 1: 20,000- 50,000 births [2,3]. FGFR3 is the only gene known to cause TD [2]. It was mapped to chromosome 4p16.3, and consisted of 19 exons that spanned over 16.5kb [2,3]. While majority of them are not compatible with life and die in utero or in the first 48 hours of life, some are compatible with life, with death usually occurring before the first decade of life [3,4]. We present a case that was discovered late in third trimester.

## Patient and observation

Mrs. DOJ is a 30 year old G2P1+0 primary school teacher who first presented to the clinic at 18 weeks of gestation by ultrasound scan since she was not sure of her last menstrual period (LMP). She presented to the clinic because of the gastroenteritis in pregnancy and was managed with intravenous fluids, metronidazole and haematinics. She eventually came back to the clinic to book at about 32 weeks of gestation and she claimed she has not had any antenatal care nor medications prior booking. Examination revealed a healthy-looking woman with symphysiofundal height of 32 weeks and fetus was alive. Ultrasound scan revealed a live fetus in longitudinal lie and breech presentation. Placental was antero-fundal and there was polyhydramnios (AFI of 205mm). There was gross incompatibility between the biparietal diameter and the femur length which prompted the diagnosis of some form of skeletal dysplasia. She was referred for comprehensive fetal anomaly scan. The repeat scan showed remarkable lordosis and the skull had a clover leaf appearance. All the long bones were significantly shortened. The measured parameters showed biparietal diameter (BPD) to be 78mm, corresponding to 30 weeks 4 days and femur length to be 22mm, corresponding to 16 weeks 1 day. The estimated fetal weight was 834g. Polyhydramnios was still present and single pocket of amniotic fluid measured 10cm.

**Figure 1:**
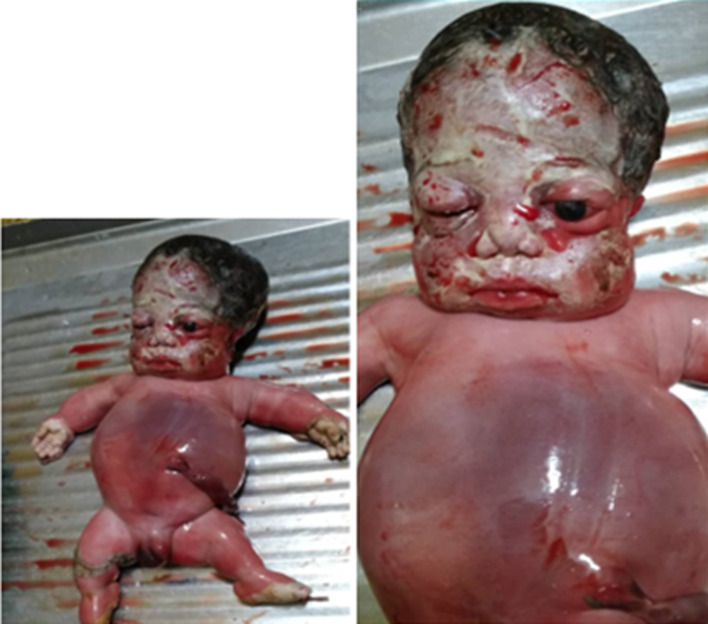
showing a macerated still birth with macrocephaly with frontal bossing, micromelia and distended stomach

**Figure 2:**
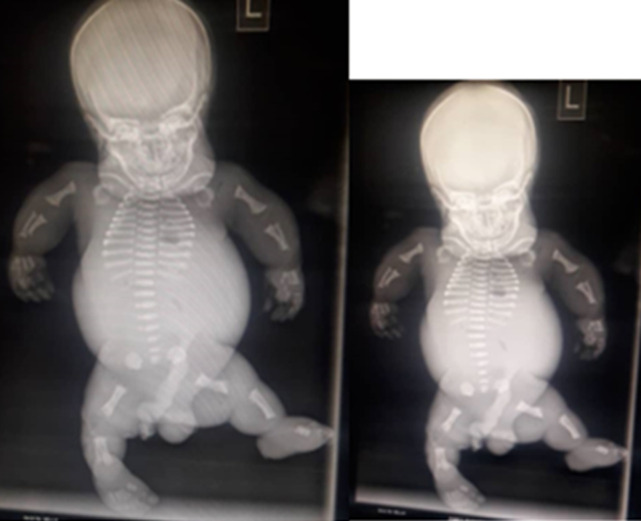
showing a babygram. There is macrocephaly with short straightened limbs, horizontal ribs and narrow chest. The iliac bones and the scapulae are also hypoplastic

A diagnosis of generalized skeletal dysplasia, thanatophoric dysplasia was made. Based on the ultrasound scan (USS) result she was counselled for termination of pregnancy which she declined. She however re-presented to the clinic at about 39 weeks of gestation with a satisfactory state of health. Abdominal examination showed a symphysiofundal height (SFH) of 37cm. Abdominal examination revealed a baby in breech presentation. The fetal heart was not heard which was confirmed by USS. She was scheduled for induction of labour which she consented to. Cervical ripening with Foleys catheter was done followed by induction of labour with misoprostol. Polyhydramnios was confirmed during artificial rupture of membranes; liquor was pot-wine coloured and the fetus was eventually expelled by breech delivery with a weight of 2.9kg. Thebaby was macerated with micromelia and the face had striking odd facie with dilated sutures and disproportionately big clover-leaf -shaped head, a short neck and mild proptosis with low set ears. Musculoskeletal system in addition showed short stubby spade like fingers and the palate was high arched. The abdomen was distended and hyperaemic with thin umbilical cord. On palpation, the abdomen was soft and; the liver was about 3cm enlarged below the costal region. The male genitalia was well developed (Figure 1). The parents rejected autopsy but accepted that the baby be donated for the teaching of students. A complete X-ray of the body of the baby (babygram) showed macrocephaly, narrowed chest with flattened ribs. All the vertebral bodies were reduced in height and appeared widened. The iliac bones were hypoplastic and all the long bones were shortened but straightened. There wasassociated metaphyseal flaring (Figure 2). A diagnosis of thanatophoric dysplasia type II was made.

## Discussion

Thanatophoric simply means death bringing which tries to explain the lethality of the disease and most of them rarely survive above the neonatal life though there are reported cases that survived beyond 9 years in type 1 [4]. The pathology is caused by a mutation of a gene responsible for production of a protein called Fibroblast Growth Factor Receptor 3 (FGFR3 gene) which is involved in the development and maintenance of bone and brain tissue. Mutations in the gene cause the protein to be overly active causing disturbances in bone growth by way of premature ossification among others [2]. There are 2 main types of TD. Type 1 is characterized by curved femora and very flat vertebral bodies; Type 2 is characterized by straight femora and taller vertebral bodies and most experience severe craniosynostosis (cloverleaf/kleeblattschädel).

The client denied use of any unprescribed medication in pregnancy until when she took ill and did not take any other medication including routine haematinics before the TD was diagnosed. It has been reported that the development of TD is a sporadic or denovo mutation and an obvious trigger factor is not known [3]. The client had an early second trimester ultrasound scan done but the pathology was not detected until the second visit to the clinic when she came to register for antenatal care. The diagnosis of TD is prenatal and this has been reported to be best in third trimester but can be done in second trimester but the accuracy can be increased with abdominal CT, which does not in any way improve the prognosis. Amniocentesis may also be done in order to have a molecular analysis [3,5,6]. The fetus was delivered dead as found in most cases likely due to severe respiratory insufficiency from a reduced thoracic capacity and hypoplastic lungs and/or respiratory failure due to brainstem compression. If delivered alive, supportive treatment in neonatal intensive care unit is always necessary as they usually die within hours or days due to respiratory insufficiency [6]. Those who have survived for about a decade of life were found to have a genetic mutation that made the TD less lethal [4,7,8].

Thanatophoric dysplasia belongs to a family of dysplasias and all of them have the FGFR3 gene mutation but with varying extent and they therefore serve as differential diagnosis in the management. Achondroplasia is the most common non-lethal skeletal dysplasia. The distinctive clinical and radiological features allow a precise diagnosis, as there is little variability in the appearance of affected patients. Hypochondroplasia is a relatively common, milder form of achondroplasia, which varies within and between families and lacks the neurological complications often seen in achondroplasia, it is however difficult to diagnose prenatally [5,9].

## Conclusion

Although TD is autosomal dominant, majority occur sporadically and recurrence risk is low for only one previously affected fetus, early ultrasound scan might be very important in a woman who has had previous history of a child with TD to relieve parental anxiety and prevent late detection [6].
